# The Secreted Protein C10orf118 Is a New Regulator of Hyaluronan Synthesis Involved in Tumour-Stroma Cross-Talk

**DOI:** 10.3390/cancers13051105

**Published:** 2021-03-05

**Authors:** Ilaria Caon, Maria Luisa D’Angelo, Barbara Bartolini, Elena Caravà, Arianna Parnigoni, Flavia Contino, Patrizia Cancemi, Paola Moretto, Nikos K. Karamanos, Alberto Passi, Davide Vigetti, Evgenia Karousou, Manuela Viola

**Affiliations:** 1 Department of Medicine and Surgery, University of Insubria, Via J.H. Dunant 5, 21100 Varese, Italy; i.caon@uninsubria.it (I.C.); marialuisadangelo@gmail.com (M.L.D.); bartolini.barbara@gmail.com (B.B.); e.carava@uninsubria.it (E.C.); a.parnigoni@uninsubria.it (A.P.); paola.moretto@uninsubria.it (P.M.); alberto.passi@uninsubria.it (A.P.); Davide.Vigetti@uninsubria.it (D.V.); manuela.viola@uninsubria.it (M.V.); 2 Department of Biological Chemical and Pharmaceutical Sciences and Technologies (STEBICEF), University of Palermo, 90128 Palermo, Italy; flaviacontino@hotmail.com (F.C.); patrizia.cancemi@unipa.it (P.C.); 3 Centro di Oncobiologia Sperimentale (C.O.B.S.), Oncology Department, La Maddalena, 90146 Palermo, Italy; 4 Biochemical Analysis and Matrix Pathobiology Research Group, Laboratory of Biochemistry, Department of Chemistry, University of Patras, 26110 Patras, Greece; n.k.karamanos@upatras.gr

**Keywords:** hyaluronan, hyaluronan synthase 2, breast cancer, MCF-7, MDA-MB-231, estrogen receptor, golgin104, tumour microenvironment

## Abstract

**Simple Summary:**

Hyaluronan is a main glycosaminoglycan in extracellular matrix with an important role in breast cancer progression. Alterations in its synthesis and size may affect tu-mour growth and metastasis. Communication between stromal and breast cancer cells consists of the secretion of factors that provoke a series of cell signalling that influence cell fate and tis-sue microenvironment, by favouring tumour cell survival and motility. Here, we present the c10orf118 protein expressed in high amounts by breast tumour cells as a new regulator in hya-luronan synthesis. This protein is found both in Golgi and secreted in the extracellular matrix, whereas its role is still unknown. The secreted c10orf118 is found to induce hyaluronan synthase 2 in normal fibroblasts. Importantly, high expression of c10orf118 is positively correlated to pa-tient’s survival and to a low metastasis.

**Abstract:**

Interaction between cancer cells and their microenvironment is central in defining the fate of cancer development. Tumour cells secrete signals (cytokines, chemokines, growth factors) that modify the surrounding area, while the niche supplies structures and activities necessary for tumour maintenance and growth. Hyaluronan (HA) is a glycosaminoglycan that constitute cancer cell niche and is known to influence tumour functions such as proliferation, migration and neoangiogenesis. The knowledge of the factors regulating HA synthesis and size is crucial in understanding the mechanisms sustaining tumour development. Here we show that a yet uncharacterized protein secreted by breast tumour cell lines, named c10orf118 (accession number NM_018017 in NCBI/BLAST, and Q7z3E2 according to the Uniprot identifier), with a predicted length of 898 amino acids, can induce the secretion of HA by stromal fibroblasts through the up-regulation of the hyaluronan synthase 2 gene (HAS2). Intracellularly, this protein is localized in the Golgi apparatus with a possible role in vesicle maturation and transport. The expression of c10orf118 was verified in breast cancer patient specimens and was found to be associated with the presence of estrogen receptor that characterizes a good patient survival. We suggest c10orf118 as a new player that influences the HA amount in breast cancer microenvironment and is associated with low aggressiveness of cancer.

## 1. Introduction

Cancer growth is largely determined by modified communication between tumour cells and the surrounding microenvironment. The contribution of the latter to cancer progression is central and as tumour cells secrete specific signals that modify the surrounding area, similarly the opposite process takes place, with the niche producing modified structures and activities necessary to survival, growth and development of the cancer cells [1].

In this light, both the signals expressed and secreted by tumour cells and the composition of the relative niche, require the same attention in order to unravel the complex crosstalk that feeds and maintains tumour growth [2]. For instance, it is known that tumours release a series of molecules to favour angiogenesis and metastasis. Among these there are inhibitor of differentiation proteins [3], growth factors with paracrine signalling to re-program surrounding fibroblasts [4], chemokines and tumour-specific antigens that contributes to recruit and transform immune cells to sustain tumour survival [5]. In addition, the whole metabolites produced by cancer cells are today recognized as strategic players in cancer development [6]. On the other hand, the surrounding stroma must provide support, nourishment, protection from the immune system and drug activity to the cancer cells, involving a variety of molecules and metabolites [7]. The tumour surrounding stroma is mainly composed by cancer associated fibroblasts (CAFs), which may represent up to the 70% of the whole tumour volume, displaying specific features for clinical diagnosis, prognosis, cancer progression and chemotherapy resistance [8]. Concerning the tumour microenvironment, several molecules such as matrix metalloproteinases, growth factors, interleukins, chemokines, collagen and proteoglycans have been recognized as participants to the tumour-stromal interplay. In particular, hyaluronan (HA) one of the major glycosaminoglycans (GAGs) found in extracellular matrices regulates tumour cell migration and invasion in vitro, and tumour growth and progression in vivo [9,10,11,12,13,14].

Alterations of HA amount, size and expression are tightly correlated to cancer progression with specific contribution to breast cancer microenvironment remodelling [15,16]. HA is often enriched in the stroma that surrounds breast tumour thus promoting tumour cell functions, such as proliferation, and neoangiogenesis. Breast tumour cells express low levels of the enzymes that synthesize HA, i.e., HA synthase (HASs), but express high amounts of the degrading enzymes hyaluronidases (HYALs) [17,18]. Thus, the increment in HA synthesis by stromal cells may be the consequence of paracrine signalling from tumour to stroma [19]. Moreover, the degradation of HA by HYALs of tumour cells, forms fragments of low molecular weight HA, which, in turn, modify the ECM and eventually accelerate the tumour neovascularisation and enhances metastasis. This “cancerization” of the stroma following by perturbation of the HA metabolism has been shown to be associated to diminished survival in breast cancer [20]. The enrichment in HA can be due also to cytokines and growth factors activity. In fact, there are evidences of the action of transforming growth factor β (TGFβ) in inducing HA synthesis in mammary epithelial cells driving the epithelial-mesenchymal transition characterizing cancer progression [21]. Interestingly, HA biosynthesis and catabolism are able to regulate the metabolism and therefore the behaviour of cancer cells, influencing the hexosamine biosynthetic pathway and therefore the availability of energy substrates [22,23].

Alterations in ECM composition, especially in HA deposition and HA related enzymes synthesis, are peculiar to understand tumour-stroma interactions and to study the possible mechanisms involved in tumorigenesis.

Therefore, we aimed to analyse the metabolite(s) produced by cancer cell lines to verify their potentiality in regulating HA production by normal dermal fibroblasts, which are the most abundant cell type in the tumour stroma [24].

Using proteomic techniques, we found that the breast cancer cell line 8701-BC (derived from a primary ductal infiltrating carcinoma) secretes considerable amounts of the uncharacterized protein c10orf118, known also as coiled-coil domain-containing protein 186 or Q7z3E2. Although the lack of a well-documented literature about the functions of this protein (especially in breast cancer) some papers described c10orf118 as a golgin. As for all the golgins, c10orf118 is a large protein of more than 500 residues (898 aa), interacts with a member of the Rab proteins (Rab2), localizes in the Trans Golgi Network (TGN) and contains coiled-coil motifs. All these features brought Gillingham and colleagues to name c10orf118 golgin104 in humans and flies [25]. Moreover, similarly to golgins that are involved in vesicles tethering and transport [26], c10orf118 seems to regulate dense core vesicles maturation and to control the post-Golgi retention of cargos in neurons [27].

With the present study we demonstrate that in vitro c10orf118 induced HA secretion by acting on the up-regulation of the HAS2 gene in fibroblasts and was also identified in tissue specimen of patients diagnosed with breast cancer. Interestingly, the c10orf118 gene expression was associated with a higher level of expression of the estrogen receptor, whose presence correlates with positive outcome in cancer [28,29].

Hence, we propose that c10orf118 is a novel player in the crosstalk between breast cancer cells and the stromal microenvironment and that its specific secretion may be related to the different aggressiveness of the tumour. Further investigations will be necessary to unveil the autocrine and paracrine in vivo role of c10orf118 protein on HA synthesis and metabolism.

## 2. Results

### 2.1. A Soluble Factor in 8701-BC Cells Conditioned Medium Induces Expression of HAS2 in NHDF

In preliminary experiments, we aimed to verify the potential ability of cancer cells to modulate HA expression pathway. Therefore, we cultured the epithelial breast cancer cell line 8701-BC, an estrogen receptor negative (ER-) with intermediate aggressiveness cell line [30] derived from a primary ductal infiltrating carcinoma [31], in their specific M199 medium. The choice of this cell line was motivated by the fact that it does not require supplementation of serum in the culture media for optimal growth and function, thus the conditioned media (CM) isolated from the cells is the cleanest system for the identification through mass spectrometry of possible secreted factors that are not “masked” by the serum concentration.

**Figure 1:**
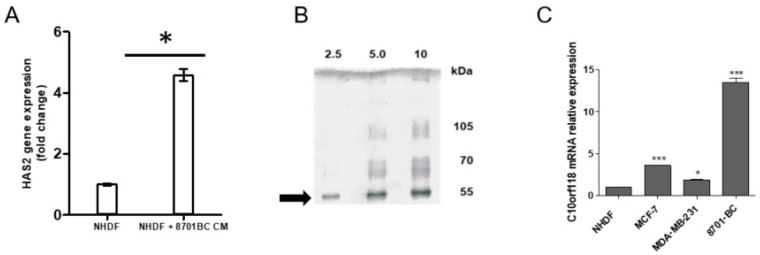
The 8701-BC cells express a soluble factor that is responsible of HAS2 induction in NHDF. (**A**) Quantitative RT-PCR analysis of HAS2 gene in NHDF and NHDF cultured in 8701-BC 48 h CM. The results are expressed as fold change with respect to NHDF cultured in RPMI1640 and each bar represents mean ± S.D. of two independent experiments. * *p* < 0.05. (**B**) SDS PAGE after Blue Coomassie staining of different amounts in term of total proteins (2.5, 5, 10 μg respectively) of 8701-BC 48 h CM. Bands at MW of approximately 55 kDa were excised and analyzed by MALDI-TOF as reported in Material and Methods. (**C**) Quantitative RT-PCR analysis of the c10orf118 (Q7z3E2) gene expression level in different breast cancer cell lines (MCF-7, MDA-MB-231 and 8701-BC), expressed as fold change with respect to NHDF. Bars represent mean ± S.D. of triplicate samples. * *p* < 0.05, *** *p* < 0.001.

Once at confluence, the CM was collected and used for the treatment of human normal dermal fibroblasts (NHDF). An induction of HAS2 gene was observed in NHDF cultured for 72 h in the presence of 8701-BC CM (Figure 1A). We focused on the analysis of HAS2 as it shows the highest HA production in tissues [32].

To identify the potential soluble factor(s) in the medium responsible for the induction of HAS2, SDS-PAGE analysis of the total proteins of the CM of 8701-BC cells in the absence of FBS was performed. Interestingly, SDS-PAGE revealed the presence of several bands. The most defined one at around 55 kDa was excised (Figure 1B) and analysed by MALDI-TOF and the Mascot Search by Matrix Science, as described in Materials and Methods. Results showed peptides that matched for the c10orf118 protein (see Supplementary Figure S1A,B). This protein of 898 amino acids and with a predicted full-length molecular weight of 104 kDa has the accession number NM_018017 in NCBI/BLAST, corresponds to Q7z3E2 according to the Uniprot identifier and has alternative names such as “coiled-coil domain containing 186 (CCDC186)” and “CTCL-tumour associated antigen”. Interestingly, a band with a size compatible with the full-length protein was detectable in the SDS-PAGE of the 8701-BC CM (Figure 1B). According to the Ensembl database, the CCDC186 protein presents different isoforms with different predicted molecular size. Beside the full-length protein (898 aa, 104 kDa), two additional isoforms of 436 aa (51 kDa) and 211 aa (24 kDa) are reported as summarized in Supplementary Table S1. Because of the limited information available on this protein in the literature, further bioinformatics research was performed. There are six different gene variants but only four of them express the protein (see Supplementary Table S1). Based on these results, antibodies and primers for further studies were chosen on the gene and protein sequences of the CCDC186-207 and CCDC186-204 variants, which represent the full-length and the second in length isoforms, respectively.

Phyre2 program was used for the prediction of the protein 3D-structure, as described before [33]. With 79% of residues modelled at >90% confidence, the 3D-structure showed the presence of many alpha-helix as secondary structures (see Supplementary Figure S1C), which are typical of coiled coil domain proteins. Computational programs and experimental techniques were used for the study of the localization of c10orf118 protein. Using the comPPI database (http://ComPPI.LinkGroup.hu, accessed date 30 October 2016), the major localization of c10orf118 (searched as Q7z3E2) was predicted in the secretory pathway of Golgi apparatus and in the cytosol, whereas in the nucleus c10orf118 was predicted with minor localization score (See Supplementary Table S2).

To verify the expression of c10orf118 gene as specific product of breast cancer cells, two more conventional breast cancer cell lines and the stromal NHDF as control were analysed by quantitative RT-PCR. Expression of c10orf118 gene was found more pronounced in the breast cancer cells, with respect to stromal cells (Figure 1C). The highest rate of expression was found in the less aggressive 8701-BC and MCF-7 cell lines, in comparison to MDA-MB-231 that is a high aggressive breast cancer cell line [30,34,35].

### 2.2. C10orf118 Is Found Both in Cell Lysate and Secreted Media of Different Breast Cancer Cell Lines

**Figure 2:**
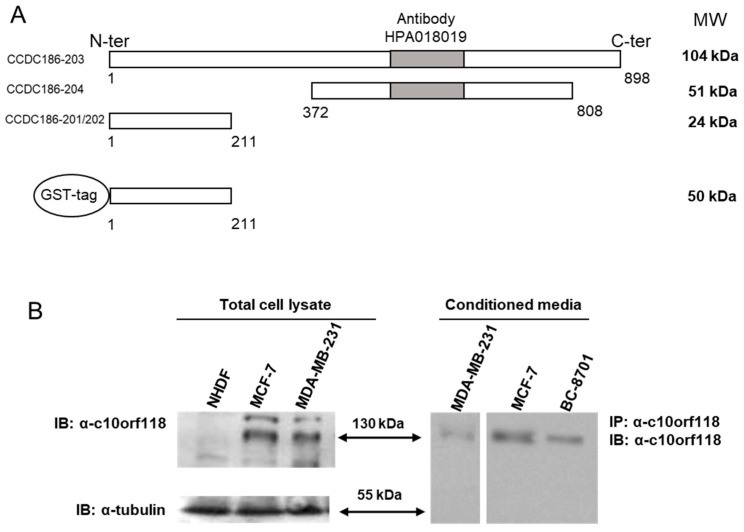
C10orf118 is found both in cell lysate and secreted media of different breast cancer cell lines. (**A**) Schematic view of the different isoforms of c10orf118 protein as reported by ensembl.org database, reporting the amino acid position and the molecular weight. Grey box depicts the region recognized by the antibody HPA018019 (Sigma Aldrich) and schematic view of the c10orf118 human recombinant peptide bearing the first 211 amino acids and a GST tag at N-terminal position. (**B**) Left panel, immunoblot of c10orf118 (full size, arrow) and tubulin in 30 μg of total proteins of cell lysate from NHDF, MCF-7 and MDA-MB-231 cells, respectively; right panel, immunoprecipitation and immunoblot of c10orf118 (full size, arrow) in 25 μL CM from culture of MDA-MB-231 and MCF-7.

To verify whether c10orf118 gene expression was accompanied to increased protein level, evaluation of the protein both in cell lysates and CM of cancer cell lines was assessed by means of an anti-c10orf118 antibody (HPA018019, Sigma Aldrich, Milano, Italy), directed against a 131-residue sequence within the central portion of the protein (Figure 2A).

Despite in silico prediction analyses describe c10orf118 as a 104 kDa full-size protein, western blot experiments performed both in cell lysates and CM using the anti-c10orf118 antibody (HPA018019) revealed a band around 130 kDa (Figure 2B) which is also in accordance with recent data in literature [36]. This suggests a possible pattern of glycosylation or other post translational modifications on the full-size isoform. Additionally, using this antibody we were not able to see a 55 kDa band in 8701-BC cells extracts, as expected by Figure 1B, which could probably correspond to the 51 kDa predicted size (Figure 2A) According to the gene expression, the synthesis of c10orf118 full size was significantly elevated in breast cancer cells with respect to normal fibroblasts. The non-aggressive cell line MCF-7 showed the highest rate of synthesized protein in both cell lysate and CM (Figure 2B), whereas stromal cells showed minimum amount of protein in cell lysate. In NHDF the presence of the protein was not checked in CM as both gene expression (Figure 1C) and protein content in cell lysate (Figure 2B, left panel) were in minute amounts.

### 2.3. Endogenous c10orf118 Localizes in the Golgi Apparatus in MCF-7 Cells

**Figure 3:**
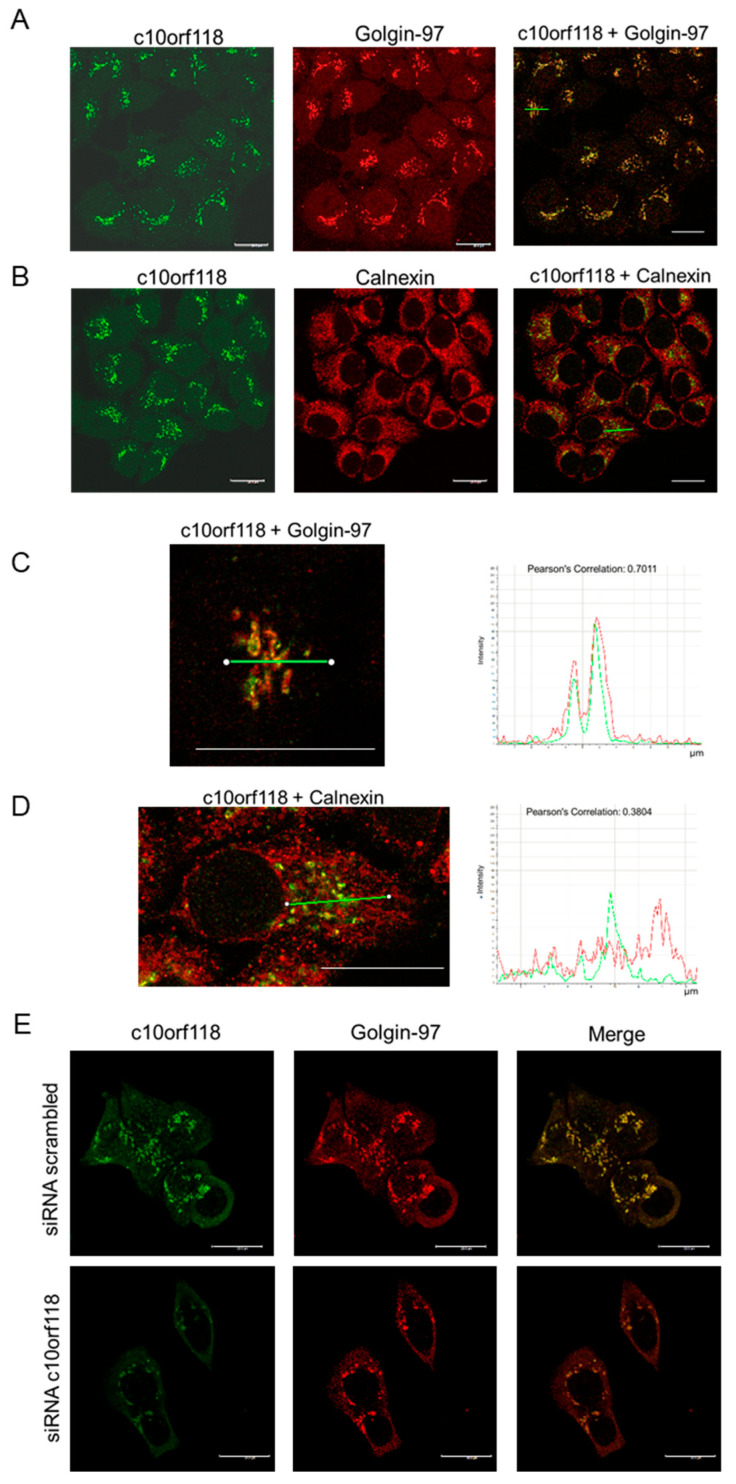
Endogenous c10orf118 localizes in the Golgi apparatus in MCF-7 cells. (**A**) Confocal microscopy images of MCF-7 cells stained for c10orf118 (green) and Golgin-97 (red), a Golgi apparatus marker. (**B**) Confocal microscopy images of MCF-7 cells stained for c10orf118 (green) and calnexin (red), a specific endoplasmic reticulum marker. (**C**) Line-scan of the staining performed in the section marked with a green line in the merged view and relative quantification. Overlap of the two signals is apparent. (**D**) Line-scan of the staining in the section marked with a green line in the merged view and relative quantification. (**E**) Confocal microscopy images of MCF-7 cells nucleofected with 30 nmol of scrambled siRNA and siRNA against c10orf118 for 48 h and stained with antibody against c10orf118 (green) or Golgin-97 (red). White bars, 20 µm.

Based on the in silico studies presented in Supplementary Table S2, we sought to explore the localization of the c10orf118 protein within the cell by using confocal fluorescence microscopy. MCF-7 cells were fixed and stained with antibodies against c10orf118 and either Golgin-97, which is a specific Golgi apparatus protein (Figure 3A), or calnexin, a membrane protein of the endoplasmic reticulum (ER) (Figure 3B). Although the primary sequence of c10orf118 contains a KDEL signal at position 677–680, compatible with the retention in the ER, confocal images revealed a preferential localization in the Golgi apparatus (Pearson’s Correlation = 0.7011 and 0.3804 in Golgi and ER, respectively). This result was also supported by the high-resolution line-scan performed on Z stacks images with thickness of ~0.25 µm (Figure 3C,D). As a control, MCF-7 were nucleofected for 48 h with 30 nmol of siRNA against c10orf118 or scrambled siRNA, plated on coverslips, fixed and stained with antibodies against c10orf118 and Golgin-97. The silencing of c10orf118 showed a weaker (green) signal in the Golgi apparatus of MCF-7 cells stained with the antibody against c10orf118, confirming its subcellular localization (Figure 3E). All the images were taken with a 63X objective using a Leica TCS SP5 instrument.

### 2.4. Effects of C10orf118 Silencing in MCF-7 Cells

**Figure 4:**
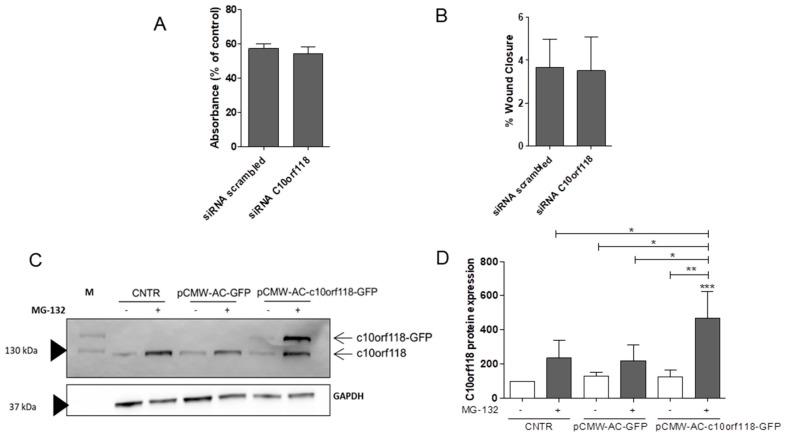
Role of C10orf118 in MCF-7 cell function. (**A**) MTT assay of MCF-7 cells nucleofected with 30 pmol of a siRNA against c10orf118 or a scrambled siRNA. Data are expressed as percentage of control ± S.D. of three independent experiments. (**B**) Wound healing assay performed in MCF-7 cells nucleofected with 30 pmol of siRNA against c10orf118 or scrambled control. The graph represents the percentage of wound closure ± S.D after 24 h from the initial scratch of four independent experiments. Images were analysed using the software tool TScratch. (**C**) Western blot of 30 µg of MCF-7 cells lysates nucleofected for 48 h with an overexpressing vector for c10orf118 (pCMW-AC-c10orf118-GFP) or the relative empty vector (pCMW-AC-GFP) and treated with 5 µM MG-132 for 24 h. M = protein marker. The images report a representative immunoblot for c10orf118 and GAPDH and (**D**) the relative quantification. The analysis was performed measuring the optical density of the bands and values are expressed as percentage variation of the control ± S.D. of four independent experiments. * *p* < 0.05, ** *p* < 0.01, and *** *p* < 0.001.

**Figure 5:**
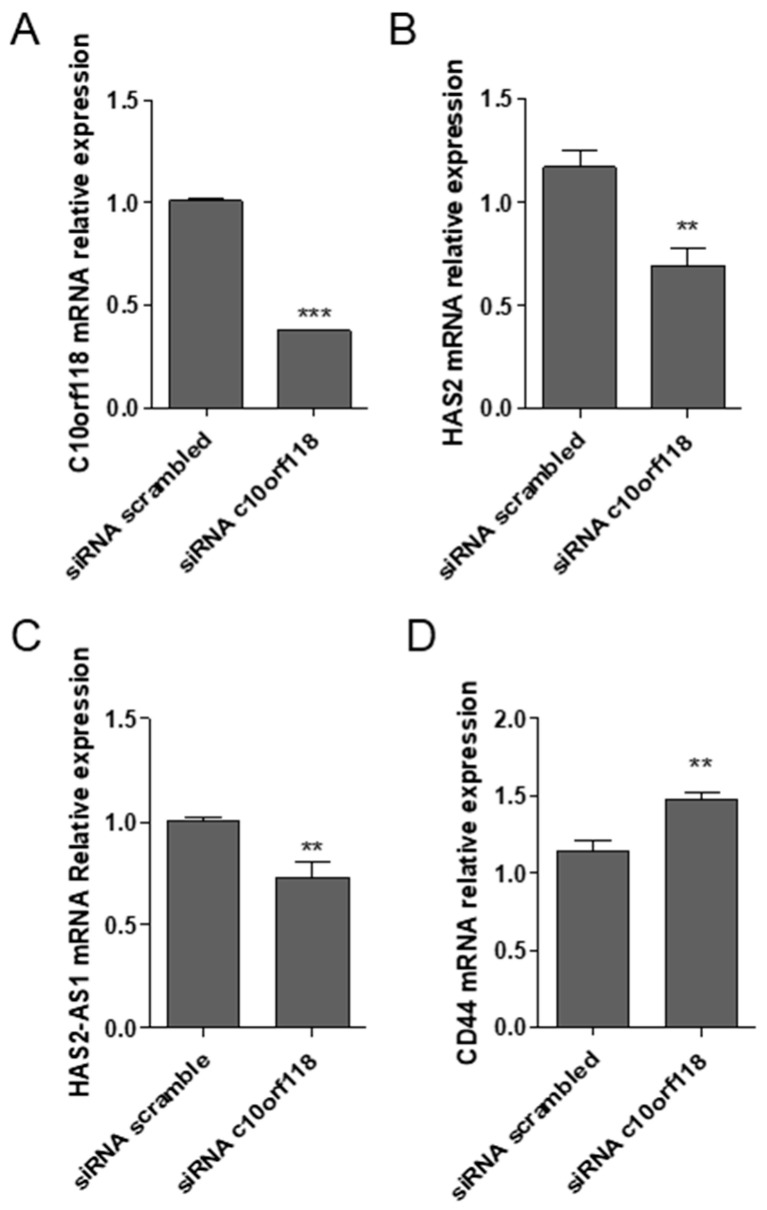
c10orf118 silencing influences HA-related genes expression in MCF-7. Quantitative RT-PCR analyses of MCF-7 cells nucleofected for 48 h with 30 pmol of c10orf118 siRNA or a scrambled siRNA. The graphs represent the mRNA levels of (**A**) c10orf118, (**B**) HAS2, (**C**) the long non-coding RNA HAS2-AS1 and (**D**) the HA receptor CD44. Data are represented as mean ± S.D. of six independent experiments. ** *p* < 0.01, and *** *p* < 0.001.

Considering the lack of information about the functions of c10orf118 in cancer, we silenced c10orf118 in MCF-7 cells by siRNA for 48 h and performed functional assays aimed to evaluate cell viability and migration. As shown in Figure 4, no significant differences were detected in cell viability (Figure 4A) and migration (Figure 4B) upon c10orf118 knockdown. Figure 4C shows that the protein is degraded by the ubiquitin-proteasome system. The treatment with the 26S proteasome inhibitor MG-132 increased c10orf118 protein amount at basal levels (bands at 130 kDa) and after the transfection of a pCMW-AC-c10orf118-GFP overexpressing vector (bands at ~160 kDa). On the contrary, the overexpression of the c10orf118-GFP fusion protein alone without the treatment with MG-132 failed, as demonstrated by a weak intensity band with a molecular weight of approximately 160 kDa (Figure 5C,D), most probably because of the immediate degradation through the ubiquitin-proteasome system. Considering these results, we decided to modulate c10orf118 expression by siRNA silencing.

### 2.5. c10orf118 Silencing Influences HA-Related Genes Expression in MCF-7

Changes in matrix deposition and alterations of genes involved in ECM synthesis, including the ones involved in HA metabolism, are key features in cancer. Here we show that the knockdown of c10orf118 by the nucleofection of 30 nmol siRNA reduced the mRNA levels of HAS2 and its epigenetic regulator HAS2-AS1 [37,38] (Figure 5B,C), whereas increased the levels of the HA receptor CD44 (Figure 5D) with respect to MCF-7 cells transfected with 30 nmol of a scrambled siRNA. C10orf118 silencing efficiency was ~65% (Figure 5A). Thus, there is an autocrine effect of c10orf118 protein on key enzymes and receptors related to HA.

### 2.6. Specificity of c10orf118 Protein Action in Inducing HA Synthesis by Stromal Cells

**Figure 6:**
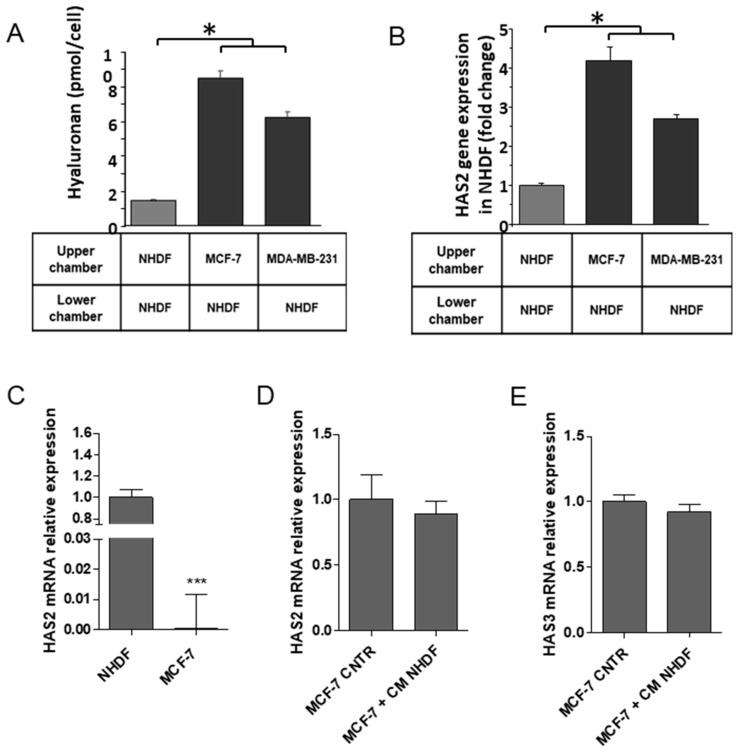
Effects of fibroblasts/breast cancer cells co-culture on HA production and HAS2 gene expression. (**A**) HA secretion detected by HPLC in the culture medium and (**B**) HAS2 expression in NHDF co-cultured with MCF-7 and MDA-MB-231 cells (see Materials and Methods). Bars represent mean ± S.D. of triplicate samples. (**C**) Quantitative RT-PCR of HAS2 expression in NHDF and MCF-7 cells. (**D**) Relative gene expression of HAS2 and (**E**) HAS3 in MCF-7 cells grown for 24 h in the absence (CNTR) or presence of NHDF conditioned media (+ CM NHDF). * *p* < 0.05, *** *p* < 0.001.

To investigate the effect of the secreted c10orf118 protein on HA synthesis, we set up an in vitro model of co-culture of NHDF cells with either MCF-7 or MDA-MB-231 cell lines (see Materials and Methods). We found a significant increased amount of secreted HA and increased gene expression of stromal HAS2 in both systems (Figure 6A,B). This result was specifically marked in the co-culture with MCF-7 cells. As reported in Figure 6C, NHDF express a huge amount of HAS2 mRNA with respect to MCF-7. Moreover, the treatment of MCF-7 cells with NHDF CM did not alter neither HAS2 nor HAS3 expression (Figure 6D,E), suggesting that the HA measured in Figure 6A is predominantly produced by NHDF. HAS1 and HAS3 expression in the co-culture system is not reported as these synthases are weakly expressed (data not shown). This data is supported by several papers [19,39,40] and it is known from the literature that HAS1 has a very low enzymatic activity [39,40].

**Figure 7:**
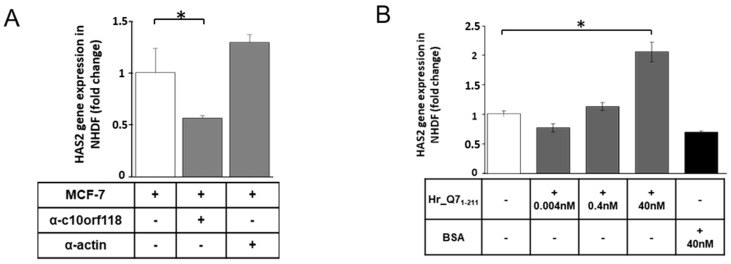
Specificity of c10orf118 effect on HAS2 synthesis by NHDF. (**A**) Quantitative RT-PCR analysis of HAS2 expression by NHDF co-cultured with MCF-7 alone or in the presence of an anti-c10orf118 polyclonal antibody or an anti-α-actin antibody as negative control. Each bar represents mean ± S.D. of triplicates. * *p* < 0.05. (**B**) Quantitative RT-PCR analysis of HAS2 expression by NHDF in the presence of increasing concentrations of a recombinant c10orf118 peptide (Hr_Q71-211). Bars represent mean ± S.D. of three replicates. * *p* < 0.05.

To further investigate whether the increment of HA secretion and HAS2 expression by NHDF was specifically due to the c10orf118 secreted into the medium by breast cancer cells, CM of MCF-7 was incubated with 4 μg/mL of anti-c10orf118 antibody (HPA018019) and subsequently used for NHDF treatment (Figure 7A). The anti-actin antibody was used in parallel as negative control. Real-time PCR results showed that neutralization of c10orf118 protein in MCF-7 CM diminished the induction of HAS2, confirming that this protein secreted by breast cancer cells plays a key role in the stimulation of HA synthesis in stromal cells (Figure 7A). To further confirm this data, NHDF were treated with a commercial recombinant peptide for c10orf118, named Hr_Q71-211 (Figure 7B), corresponding to the first 211 residues of the c10orf118 and fused with a GST-tag at N-terminus (Figure 2A), in three different concentrations: 0.004 nM, 0.4 nM and 40 nM. Induction of HAS2 expression was significantly increased when cells were treated with 40 nM of recombinant protein, demonstrating that c10orf118 induces HAS2 (Figure 7B) in a dose dependent manner.

Thus, we hypothesize that there is a consequential paracrine effect between secretion of c10orf118 by breast cancer cells and increment of HA by stromal cells with a mechanism that is still to be explored.

### 2.7. C10orf118 Is Detected in Breast Cancer Cell Tissues and Is Estimated to Associate with Estrogen Receptor (ER) Expression

The secretion of the c10orf118 in culture cell media suggests a role of this yet uncharacterized protein in influencing the surrounding environment. This is one of the peculiar features of cancer cells, which aim to modify their microenvironment in order to proliferate. To prove our findings in vivo, we verified the c10orf118 gene and protein expression in a cohort of 40 patients with ductal breast cancer (G2/G3) by quantitative real time PCR and western blot analyses. Results revealed that both gene and protein expression were highly variable among patients (see Supplementary Figure S2).

**Figure 8:**
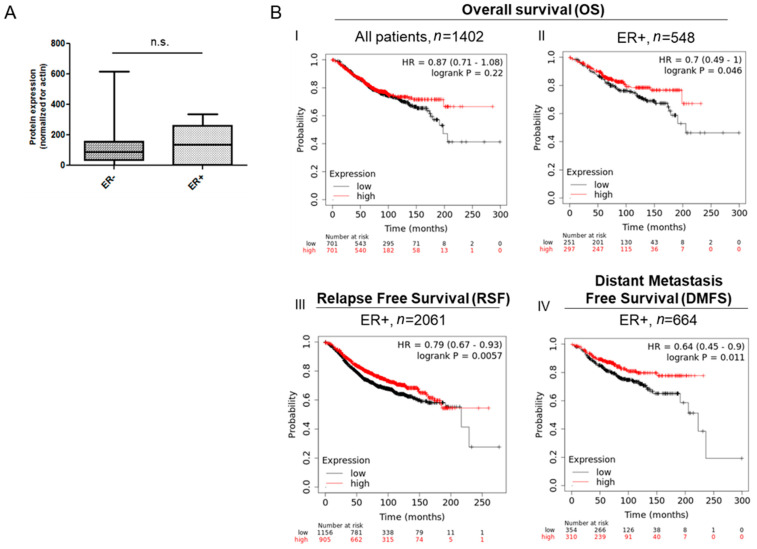
C10orf118 is highly expressed in breast tumour tissues that express ER and its level of expression is correlated with higher overall survival. (**A**) Quantitative analysis of c10orf118 expression normalized to actin as obtained from immunoblot of cancer patient specimens. (**B**) Kaplan-Meier survival plots obtained by http://kmplot.com/analysis/ (accessed date 20 January 2021) to evaluate the overall survival (OS), relapse free survival (RFS) and distant metastasis free survival (DMFS) of breast cancer patients in the database expressing high (red line) and low (black line) levels of the c10orf118 mRNA.

To evaluate the possible prognostic role of c10orf118, we used the Kaplan Meier-Plotter tool [41] analysing survival data, evaluated as Overall Survival (OS), Relapse Free Survival (RFS) and Distant Metastasis Free Survival (DMFS) of 3951 breast cancer patients. The survival analyses were performed by using the Affymetrix ID probe 219844_at and patients were split into two groups according to the best cut-off values of the expression values. Better values of overall survival (OS), relapse free survival (RFS) and distant metastasis free survival (DMFS) were associated with the higher expression of the c10orf118 protein for all patients (Figure 8A panel I, not shown for RFS and DMSF). Intriguingly, significant association of c10orf118 with survival data (OS, RSF and DMSF) were recorded in the ER+ patients (Figure 8B panel II, III and IV, respectively) compared to all patients (data not shown). Results obtained exclusively from ER- patients are not presented as they showed a low number of patients and a relatively high number of *p*-value. The improved survival associated with higher c10orf118 expression was also demonstrated in terms of hazard ratio (HR < 1.00). Based on these results, analysed patients were split into two different groups, according to the expression of the ER. Higher levels of c10orf118 were detected in the ER+ specimens, whereas the ER- group displayed a general lower expression although with a high variability (Figure 8B).

Thus, c10orf118 could be considered a candidate of prognostic marker for ER+ tumours and it is likely that its higher expression is associated with increased probability of survival. However, further in vitro and ex vivo studies should be performed to verify this hypothesis.

## 3. Discussion

In this study, we present for a first time a novel breast cancer secreted protein able to modulate HA synthesis by stromal cells, taking part in tumour matrix remodelling. This protein, known as c10orf118, Q7z3E2, golgin104 or coiled-coil domain containing 186, is secreted by different breast cancer cell lines and is specifically able to induce HAS2 expression and HA synthesis by fibroblasts.

Reciprocal communication between tumour and stroma through factors secreted by tumour cells enables transmittance of signals to host cells that alter the tumour ECM microenvironment specifically. ECM composition can modulate tumour cell functions and signalling, eventually favouring the angiogenesis and diffusion of the metastasis. In fact, ECM and its components are believed to be dynamic players in remodelling and influencing cell behaviour, as it has been highlighted in several reports [42,43]. The tight interaction between cancer cells and their microenvironment at the biochemical level is an emerging area of research. Among the other ECM components, the polysaccharide HA plays a central role, being generally implicated in tissue turnover like wound healing, embryogenesis, neointima formation and neoplasia [16] and having been linked to the determination of tumour malignancy through the alteration of signalling pathways [14]. The HA enriched matrix alters tumour microenvironment influencing tissue hydration and the osmotic balance. The newly secreted HA interacts with specific receptors (i.e., CD44 and RHAMM) and activates signal transduction pathways that promote the malignant phenotype [44,45]. The deposition of HA within tumour stroma leads to a tumour-associated fibrosis, which contributes to cancer initiation and progression [46]. One of the peculiar features of this system is the fact that HA increment in the stroma is only partially due to the cancer cells secretion with a significant contribution by CAFs, a heterogeneous mixture of multiple resident fibroblast subtypes and infiltrated circulating mesenchymal cells, which facilitate the conversion of pre-malignant epithelial cells into tumour. Under the influence of the newly secreted HA, CAFs migrate close to the tumour cells and activate an autocrine production of HA which stimulates the migration of the HA-binding tumour subpopulation [47,48]. Although the switch from normal to activated fibroblasts needs further investigations, emerging studies report that it can be mediated by epigenetic modifications [49]. Since the synthesis of HA can be epigenetically regulated [50], we do not exclude that our protein could contribute to activate fibroblasts through the deposition of HA and epigenetic modifications.

Unexpectedly, HA has also recently been implicated as a stromal tumour-suppressing factor [51,52]. This feature is especially linked to HA degradation and the polymer size. Low molecular weight HA (LMW-HA) fragments show pro-tumorigenic properties in breast cancer [53], while high molecular weight HA (HMW-HA) chains display tumour suppressive and anti-inflammatory features [15]. The naked mole rat show incredible tumour-resistance properties due to its ability to synthesize HMW-HA chains and the resistance of fibroblast to oncogenic transformation [51]. These animal’s tissues contain larger HA polymers and less detectable fragmentation than mouse tissues. HA-mediated tumour resistance of the naked mole rat is attributed to the ability of high molecular weight HA to hyper-sensitize cells to contact inhibition and induce cell cycle arrest via p16 [54]. A number of studies showed that an over production of HA by itself does not promote an aggressive tumour phenotype and can even be tumour-suppressing blocking cell cycle transition from G1 to S phase [55].

The general mechanisms through which the cells regulate the amount and the size of HA comprise the expression and activity of the HA synthases (HASs), hyaluronidases and the binding and internalization to specific receptors as CD44 and RHAMM [14].

Hyaluronan synthase 2 (HAS2) is the HA synthesizing enzyme whose action is mostly implicated in epithelial-mesenchymal transition (EMT) [32,56], therefore the fine knowledge of the mechanism regulating its expression is pivotal to understand the development of cancer and to potentially address it with therapeutic treatment.

HAS2 expression is regulated in several fashion [57], either by secreted factors like TGFβ, interleukin-1β, fibroblast growth factor-2 (FGF-2), platelet-derived growth factor (PDGF), keratinocyte growth factor (KGF), epidermal growth factor (EGF) [58] or by post translational modification as ubiquitination, O-GlcNAcylation, phosphorylation [37,59,60], by epigenetic events [50] and other potential molecule(s) [61] secreted by tumour cells may influence its activity. Indeed, we found that cancer cell line CM is a source of an uncharacterized protein that can influence HAS2 expression.

The c10orf118 protein was quantitatively found both in the cell lysate and secreted in medium by breast cancer cell lines in different amounts and with different molecular size. Besides the full-length protein found in lysates and in the CM of MCF-7 and MDA-MB-231 cells, we could detect both the full-size protein and processed fragments in the CM of 8701-BC cells. Although *in silico* analyses assign to the full-length isoform a molecular weight of 104 kDa, our experimental data demonstrate that c10orf118 is a 130 kDa protein, which probably undergoes post translational modifications. In fact, on line prediction tools report that c10orf118 displays ubiquitination (http://www.jci-bioinfo.cn/iUbiq-Lys, accessed date 15 June 2020), N-acetylation (http://www.cbs.dtu.dk/services/NetAcet/, https://www.uniprot.org/, accessed date 15 June 2020) and phosphorylation (https://www.uniprot.org/, accessed date 15 June 2020) sites. Additionally, results here demonstrated an increase of c10orf118 protein when the proteasome was inhibited, suggesting that the protein is ubiquitinated. The type of ubiquitination, such as poly- or multi- ubiquitination, should be further explored in order to discover the role that this modification plays on c10orf118 function.

The incubation of MCF-7 cell with an antibody directed against the central domain of c10orf118, (which is common to both the full size and the 50-kDa fragment), inhibited HAS2 expression by conditioned NHDF. Nevertheless, the induction of HAS2 was obtained via stimulation with a peptide bearing the first 211 amino acids, a region found in the full size but not in the 50-kDa fragment. Although we cannot exclude the presence of small amount (undetectable by the antibody) of the processed form in the medium of both MCF-7 and MDA-MB-231 cells, our data point at a biological function for the full size c10orf118 protein, with the 50 kDa fragment that may be a degradation product, specifically produced by cell line(s) (i.e., 8701-BC).

Although the highly deregulated expression of proteins by cancer cells is a well-known mechanism, the c10orf118 is not yet described in terms of its potential effect on tumour development. Only one report is available to date [62], where authors, using serological identification of antigens recombinant expression cloning (SEREX), demonstrated the presence of the c10orf118 antigen in the sera of patients with cutaneous T-cell lymphoma and, interestingly, also in serum of a control, albeit without addressing its role and activity. In our model, the modulation of c10orf118 did not influence directly MCF-7 cells viability or migration, probably because of the functional redundancy between the golgins [63]. However, c10orf118 silencing modified the expression of HAS2 and its epigenetic regulator HAS2-AS1 [37,38], as well as the levels of the HA receptor CD44, suggesting a specific effect on HA metabolizing genes rather than the behaviour of breast cancer cells. The c10orf118 may therefore represent a secreted marker of cancer cells and, although its cancer specificity needs to be further investigated, we describe a first peculiar role in stimulating HA synthesis by breast cancer cells. To verify this hypothesis, our intent is to perform cell functional and gene expression assays in other breast cancer cell lines, such as the triple negative MDA-MB-231 or MDA-MB-468.

We explored the cellular localization of c10orf118 with bioinformatics tools. Using the HUMAN PROTEIN ATLAS database, we found a plausible localization in the Golgi apparatus, in line with our findings of involvement in a secretion pathway as supported by the presence of the protein in the culture medium and by confocal microscopy analysis. The KDEL signal within its primary sequence, known as the ligand of KDEL receptor, argues for a retention of the protein in the ER compartment and indeed, we could show a partial localization of the c10orf118 in the ER. The KDEL receptor is involved in the regulation of a complex traffic between ER and Golgi, and may not exclude the trafficking to the plasma membrane and then the secretion [64]. Moreover, cancer cells often own a dysregulated metabolism, which may alter the normal distribution and secretion of the proteins. In accord with our data, Ailion et al. demonstrated that CCCP-1 (the ortholog of c10orf118 in *C. elegans*) co-localizes with Rab2 in the Trans-Golgi Network (TGN), regulating dense core vesicle maturation [65] and confirming c10orf118 involvement in the secretory pathway. Moreover, another study of Gillingham et al. conducted in drosophila S2 cells and human COS cells confirmed that c10orf118 is a golgin, which interacts with Rab 2 family members through c10orf118 C-terminal domain [25].

How this protein is secreted, recognized by fibroblasts and which possible intracellular pathways are involved in the modulation of HA synthesis is yet to be elucidated.

Screening of online databases failed to predict a network of possible interacting proteins also because the correct 3D structure is currently unknown, being only modelled by software.

The level of expression of c10orf118 by different breast cancer cell lines appears to be inversely related with the cancer aggressiveness and invasion. MDA-MB-231 cells express lower levels of c10orf118 but are more aggressive than MCF-7, which express higher amounts of c10orf118. Differently from MCF-7 and MDA-MB-231 that derive from a pleural effusion, 8701-BC originate from an infiltrating ductal carcinoma before its metastatic effusion, and showed the highest expression of c10orf118 mRNA, suggesting a possible connection with cell metastasis and c10orf118 expression. Additionally, Kaplan-Meier estimations showed a better survival of patients when c10orf118 is highly expressed. We hypothesize that the tuning of the c10orf118 expression by tumour cells is a mechanism to switch the surrounding ECM towards a more favourable composition for the growth of the cancer itself but not for the metastasis.

Considering the role of HA in tumour progression, it is known that MDA-MB-231 cells synthesize constitutively high amounts of HA [17]. These cells may already rely on an efficient HAS expression level to influence the surrounding niche that may not need to be further enhanced. In contrast, MCF-7 cells synthesize a lower amount of HA [66], but express high levels of c10orf118, that in turn is able to alter the HA metabolism in the neighbour fibroblasts. Therefore, such cells potentially contribute to increase proliferation and/or differentiation of stromal cells through the newly deposition of HA, as these features are typically targeted by HA in the matrix [67,68].

When the level of expression of c10orf118 was evaluated in patients’ tissue, it was found a possible higher expression rate that may be correlated with the expression of the ER. However, to confirm this data the study should include a higher number of tissue specimens. It is reported that the presence of ER is associated with better prognosis in breast cancer, as the expression of the receptor increases the likelihood to respond to hormonal therapy [29]. Nevertheless, our data point out a mere association between the ER and c10orf118 expression, without further functional interactions.

Recently, a novel finding links HAS2 overexpression to altered ER signalling in ER positive MCF-7 cancer cell line [69]. MCF-7 cells overexpressing HAS2 are reported to escape the estrogen dependency ending up in a more aggressive phenotype. Because c10orf118 may play a role on the vesicle tethering and transport of proteins to the membrane or extracellularly, it would be of high interest to explore a possible correlation between c10orf118 synthesis and HAS2 overexpression and transfer to cell membrane, in a co-culture system of breast cancer cell line and stromal cells.

Moreover, the ER expression has been shown to interfere with extracellular matrix molecule pathways [70]. MCF-7 cells depleted of the ER gene, show a dramatic change in the expression of ECM molecules like metalloproteinases and their inhibitors and fibronectin and vimentin, resulting in a changed morphology, increased proliferation rate, increased motility and invasiveness and overall in epithelial-mesenchymal transition. These data underline how ER signalling pathway and expression of ECM molecules are tightly related and can cooperate in defining the fate of cancer cells and their surrounding microenvironment. In this scenario, the c10orf118 protein could be a new player in the definition of the tumour ECM niche according to its level of expression together with ER presence. Despite the major role in HA in defining the cancer niche, other glycosaminoglycans and matrix molecules have been shown to be pivotal in cancer progression [71] and we cannot exclude that the c10orf118 may also influence the expression and activity of other glycosaminoglycans or proteoglycans. The c10orf118 protein and the specific tuning of its secretion levels by cancer cells may modulate the complex scenario of the various matrix molecules expression, resulting in either accelerating the epithelial-mesenchymal transition or in protecting from faster cancerization of the tumour stroma, according to the modulation of the matrix produced either in presence or absence of ER.

The potential activity of c10orf118 on cancer progression is highlighted by analysis of cancer patients’ database. When an online Kaplan-Meier plotter [41] was used to analyse the prognostic value of our gene of interest, we found that higher expression of c10orf118 was significantly associated with a better survival in terms of OS, RFS and DMFS, in ER positive patients, suggesting the c10orf118 as apparent protective marker. The outcome on ER negative patients is less clear and this may have several explanations. First, ER- describes a very heterogeneous group in which the included patients are pooled according to the lack of a receptor, and not because of a common feature. Second, in ER negative patients the expression of c10orf118 is highly variable compared to the ones expressing ER. The lack of the receptor may not be directly linked to a diminished c10orf118 expression, and it is therefore too speculative in this case to call c10orf118 a predictive outcome marker.

In summary, we found that a still uncharacterized protein mainly expressed by breast cancer cell lines can induce HAS2 expression and HA secretion by surrounding fibroblasts and that modification of the stromal ECM can contribute to modulate cancer progression. In addition, c10orf118 expressed by tumour cells do not affect proliferation and migration, it is localised in the trans Golgi apparatus with a still unknown role. Therefore, we propose c10orf118 as a new player in the regulation of HA secretion pathway and in the crosstalk between cancer and stromal cells that could implicate the intracellular protein transport to the cell membrane or extracellularly.

## 4. Materials and Methods

### 4.1. Cell Culture and Tissue Specimens

We used the neoplastic breast cancer cell lines MCF-7 and MDA-MB-231 kindly provided by Dr. Martin Götte (University of Münster, Münster, Germany) and the 8701-BC cell line. 8701-BC epithelial cells derive from a primary ductal infiltrating carcinoma (DIC) of human breast. They possess a karyotype with 55–60 chromosomes per cell with several rearrangements, they are positive for the carcinoembryonic antigen (CEA) and tissue polypeptide antigen (TPA) and negative for estrogen receptors mRNA. Moreover, they secrete urokinase and a panel of MMPs and MMPs inhibitors (TIMP1 and TIMP2) and express some tumour-related cytokine m-RNAs (TGFb 1,-2, -3, and PTHrP). Lastly, they show an intermediate neoplastic aggressiveness [30] and a moderate invasive potential, both in vitro and in vivo [72,73]. As non-tumour cells control, we used the Normal Human Dermal Fibroblast (NHDF) obtained from Lonza (Milan, Italy).

8701-BC cells were grown in M199 medium (Lonza) with L-glutamine culture medium (ECM2001L; Euroclone, Milan, Italy), 100 U/mL penicillin + 100 µg/mL streptomycin (ECB3001D; Euroclone) and 10% FBS. For proteomic analysis 8701-BC cells were grown with no serum supplementation. All other cells were grown in RPMI1640 with stable L-glutamine culture medium (ECM2001L; Euroclone) supplemented with 10% fetal bovine serum (FBS) EU Approved (ECS0180D; Euroclone) and 100 U/mL penicillin + 100 µg/mL streptomycin (ECB3001D; Euroclone) in an atmosphere of humidified 95% air, 5% CO_2_ at 37 °C in tissue culture T 75-cm^2^ flasks.

Aliquots of breast cancer tissues were obtained during surgical intervention between 2003 and 2007 in the Breast Unit of the La Maddalena Hospital and immediately frozen at −80 °C until used. Research was carried out in compliance with the Helsinki Declaration with the written consent of the patients and with the approval of the Institutional Review Board (N 515/2008) from the La Maddalena Hospital. The patients of this study did not receive any cytotoxic/endocrine treatment prior to surgery. Diagnosis of ductal breast cancer (G2/G3) was confirmed histopathologically.

### 4.2. Isolation of a Soluble Factor from 8701-BC Cell Conditioned Medium by MALDI/MS

8701-BC cells were grown for 48 h, medium was collected, and proteins quantified with Bradford reagent. Amounts of 2.5, 5 and 10 µg of total proteins were loaded in SDS PAGE. After the incubation of the gel with a Blue Coomassie solution, the most evident band at 55 kDa was excised from the gel and analyzed by MALDI-TOF mass spectrometry [74].

Briefly, the spot from the gel was destained, reduced, alkylated, and submitted to hydrolysis with endoproteinase Glu-C (SIGMA-Aldrich, Milan, Italy). Peptide mixtures were analyzed by MALDI-TOF mass spectrometry using a Voyager DE-PRO mass spectrometer (Applied Biosystems, Monza, Italy). Samples were freeze-dried and then dissolved in 10 μL of 0.2% trifluoroacetic acid; 1 μL was mixed with 1 μL of a solution of alpha-cyano-4-hydroxycinammic acid, 10 mg/mL in acetonitrile, 0.2% trifluoroacetic acid 7:3 (*v*:*v*), and the mixture was applied onto the metallic sample plate and air dried. Mass calibration was performed using a peptide standard mixture provided by the manufacturer. All mass values are reported as monoisotopic masses, and raw data were analyzed using software provided by the manufacturer.

### 4.3. NHDF Treatment with 8701-BC Conditioned Medium

8701-BC were grown for 48 h, before use CM was centrifuged to remove cell debris and analyzed for glucose content (Ospedale di Circolo Varese). NHDF were seeded 250,000 cell/well in RPMI in a 6-well plate, at 70/80% confluence, put in quiescence for 24 h, and either treated with 8701-BC CM, or normal medium. After 72 h, NHDF cells were harvested for RNA in TRI Reagent^®^.

### 4.4. SDS-PAGE and Western Blot

Cells were grown to confluence were rinsed twice with ice-cold PBS followed by lysis in 300 µL of ice-cold RIPA buffer + Protease Inhibitors. Cell lysates were scraped on ice and the protein content was measured using Coomassie Protein Assay kit (Thermo Scientific, Milan, Italy). Aliquots containing equal protein concentration were mixed with 1 volume of reducing sample buffer, denatured and separated by SDS-PAGE, followed by transfer to nitrocellulose. Membranes were blocked with 5% BSA in Tris-buffered saline (TBS) supplemented with 0.1% Tween-20. After blocking and washing with TBS and 0.1% Tween-20 (TBS-T), the membranes were probed with anti-c10orf118 rabbit polyclonal antibody (HPA018019; dilution 1:500) (Sigma) in T-TBS containing 5% BSA over night at 4 °C. After washes with T-TBS, the membranes were incubated with HRP-conjugated anti-rabbit IgG diluted 1:10,000 (Santa Cruz Biotechnology, Milan, Italy) in 5% BSA in T-TBS for 1 h at room temperature and immunocomplexes detected by enhanced chemiluminescence (Amersham ECL Prime Western Blotting Detection Reagent, GE Healthcare, Milan, Italy) according to the manufacturer’s instruction. As a control, we used a primary polyclonal antibody against α-tubulin (goat; dilution 1:300) or polyclonal antibody against GAPDH (dilution 1:200, Santa Cruz Biotechnology) and a secondary donkey anti-goat IgG-HRP (dilution 1:20,000, Santa Cruz Biotechnology). Densitometry analysis was performed using the ImageJ Gel Analysis tool, where gel background was also removed individually for each band.

### 4.5. Immunoprecipitation

At 70–80% confluence in a T-75 flask, the CM was harvested from all the cell lines and centrifuged at 14,000× *g* for 15 min at 4 °C, to remove insoluble debris. A 4-mL aliquot was concentrated to 1 mL volume into GyroVap and desalted using PD-10 Desalting Columns (GE Healthcare). The CM were poured into 15-mL tubes, frozen and lyophilized to dryness. The samples were resuspended with 50 µL of RIPA buffer containing 1× Sigma FAST Protease Inhibitors. Samples were pre-cleared with 50% G-Sepharose beads in RIPA buffer + protease inhibitors for 2 h at 4 °C on an orbital shaker. Beads were removed by centrifugation at 10,000 rpm for 1 min. The c10orf118 antibody (2.5 µg) was added to the sample and incubated at 4 °C overnight on an orbital shaker. To collect the immunocomplex, G-Sepharose beads were added to the sample and incubated at 4 °C overnight. All immunoprecipitates were washed five times with RIPA buffer. Beads were boiled in 3× sample buffer for 5 min at 95 °C and centrifuged. Eluates were analysed with SDS-PAGE and western blot.

### 4.6. Immunofluorescence and Confocal Microscopy

For confocal microscopy, 7.5 × 10^4^ MCF-7 cells were plated onto coverslips in 12-well plate. Coverslips were washed three times with PBS and fixed for 15 min with 4% paraformaldehyde in PBS at 20 °C. Cells were then washed with PBS and permeabilized by a 15 min incubation with 0.1% Triton in PBS at room temperature (RT). Unspecific site blocking was performed for 1 h with 5% BSA in PBS-Tween-20 0.1%. After washing with PBS, coverslips were incubated with primary antibodies: anti c10orf118 polyclonal rabbit 1:200 (pa5-53704 Invitrogen, Milan, Italy), anti-golgin-97 monoclonal mouse (CDF4) 1:100 (a-21270 Invitrogen), anti-calnexin monoclonal mouse (AF18) 1:100 (ma3-027 Invitrogen) in PBS-T 1%BSA over night at 4 °C.

Subsequently, cells were washed with PBST 1%BSA and incubated with a proper secondary antibody: anti-rabbit-FITC 1:100 (sc-2012, Santa Cruz), anti-mouse-TRITC 1:100 (t4202 Sigma), 1 h 30 min RT in the dark. After washes, coverslips were mounted on glass slides using Vectashield mounting medium and acquired using a Leica TCS SP5 confocal laser scanning system (Leica Microsystems GmbH, Wetzlar, Germany) and analysed with the Leica TCS software.

### 4.7. Cell Viability Assay (MTT)

To investigate the effects of c10orf118 on cell viability, a MTT assay was performed in a 96 well plate. Briefly, 8 × 10^3^ MCF-7cells were transiently transfected with 30 nmol of a scrambled siRNA (AM4611, Invitrogen) or a siRNA against c10orf118 (S30142, Invitrogen) by nucleofection using a Nucleofector Apparatus (Amaxa, Milan, Italy) and the Cell Line Nucleofector™ Kit V (Lonza). 48 h after the transfection the medium was replaced with 200 μL of fresh culture medium supplemented with 50 μL of 5 mg/mL MTT and incubated at 37 °C for 5 h. The reaction was stopped adding 200 μL of DMSO and 25 μL of Sorensen glycine buffer per well. The plate was read at 570 nm.

### 4.8. Wound Healing Assay

To test cell migration, we performed a wound healing test. Briefly, 3 × 10^5^ MCF-7 cells were transiently transfected for 48 h with 30 nmol of a scrambled siRNA (AM4611, Invitrogen) or a siRNA against c10orf118 (S30142, Invitrogen) by nucleofection using a Nucleofector Apparatus (Amaxa) and the Cell Line Nucleofector™ Kit V (Lonza). After 24 h, four scratches per well were done with a 20-μL pipette tip. Cells were washed three times with PBS and incubated with serum deprived medium (0.2% FBS) for further 24 h. Pictures were taken through light microscopy at different time points (0 and 24 h) and analyzed using the TScratch software [75].

### 4.9. C10orf118 Overexpression

To study the function of c10orf118 in tumour crosstalk and stromal fibroblast cell interaction, the cDNA sequence corresponding to human c10orf118 open reading frame (ORF) was cloned into a pCMW-AC-GFP vector (Origene-Temaricerca, Bologna, Italy) to overexpress the protein. Transfection of MCF-7 human breast cancer cells were performed using a Nucleofector Apparatus (Amaxa) and the Cell Line Nucleofector™ Kit V (Lonza). For each transfection, 2 µg of DNA were used according to manufacturer’s recommendations (Lonza). To verify the degradation of c10orf118 protein by the ubiquitine-proteasome systems, 24 h after the transfection cells were treated with 5 µM MG-132 (Sigma) for further 24 h.

### 4.10. RNA Extraction and cDNA Synthesis

At confluence, the cells were washed twice with PBS (Euroclone), and the total RNAs were extracted by using TRI Reagent^®^. Each of the total RNAs sample was treated with 0.5 µL of RNAse Inhibitor 20 U/µL (Roche). The purity of the RNAs was verified by measurement of A260/A280 value using spectrophotometer. Total RNA was retro-transcribed using the High Capacity cDNA synthesis kit (Applied Biosystems, Monza, Italy).

### 4.11. Quantitative RT-PCR

Quantitative RT-PCR was performed by means of TaqMan technology and a Real-Time ABI Prism 7000 apparatus (Applied Biosystems). The following human Taqman gene expression assays were used: HAS2 (Hs00193435_m1), HAS3 (Hs00193436_m1), HAS2-AS1 (Hs03309447_m1), CD44 (Hs01075861_m1), C10orf118 (Hs00215984_m1) and β-actin (Hs99999903_m1) as a housekeeping gene. PCR reaction mix contained 2.5 µL of cDNA, 12.5 µL of Universal PCR Master Mix (Applied Biosystems), 1.25 µL of Assay-on-Demand primer and probe and 8.75 µL of nuclease-free water. The PCR program consisted of an initial hot start at 50 °C for 2 min, followed by 95 °C for 10 min and 45 amplification cycles (95 °C for 15 s and 60 °C for 60 s). A relative quantitative analysis was performed, using the 2-(ΔΔCt) value [76].

### 4.12. Transwell System

A transwell system with a porous (0.4 µm pore size) polycarbonate membrane filter (Costar, Corning Incorporated, Turin, Italy) and 12-well plastic tissue culture plates were used for the NHDF-tumour cell co-cultures. NHDF cells were first seeded into 12-well culture plates at a subconfluent density of 4 × 10^4^ cells/well. Then, different types of tumour cells (1 × 10^4^/well) were added to the upper chambers. The resultant three groups were as follows: (1) NHDF—NHDF control group, (2) NHDF—MCF-7 group, and (3) NHDF—MDA-MB-231 group.

### 4.13. HPLC Analysis for HA Disaccharides Determination in Culture Medium

Medium from cell cultures was frozen at −80 °C, lyophilized and treated according to [77] to digest, derivatize and separate disaccharides.

### 4.14. Neutralization of c10orf118 by a Specific Antibody in Conditioned Medium of MCF-7 Cells

MCF-7 cells were plated in T25 flask at a density of 2 × 10^6^ cells/flask. At about 70% confluence, the culture medium was removed, and cells incubated with fresh complete medium for 48 h. For abrogation of the effect of c10orf1118 protein, CM from MCF-7 cells was harvested, centrifuged to remove cell debris and incubated with gentle agitation for 1h at 37 °C with 4 µg/mL (final concentration) of anti-c10orf118 rabbit polyclonal antibody (HPA018019; Sigma). The same concentrations of α-actin (sc-1616; Santa Cruz) were used as control. NHDF cells were plated in 6-well at a density of 5 × 10^5^ cells/well. Then, 800 µL of pre-incubated CM with blocking antibodies were added. After 48h, NHDF cells were harvested for RNA in TRI Reagent^®^ to study *HAS*es gene expression.

### 4.15. Cell Treatment with c10orf118 Recombinant Protein

The recombinant protein Human c10orf118 (AAH30557.1) obtained by Abnova was produced in vitro in wheat germ expression system and consisting of the first 211 amino acids of Human c10orf118, fused with GST-tag at N-terminal (MW = 50 kDa).

NHDF cells were plated in 6-well at a density of 5 × 10^5^ cells/well. At 70–80% confluence, different concentrations of recombinant protein (4 pM-40 nM final concentration) were added to each well. The same concentrations of BSA were used as control. After 24 h, NHDF cells were harvested for RNA in TRI Reagent^®^ to study the relative expression of *HAS2* in NHDF cells.

### 4.16. Statistical Analysis

GraphPad Prism version 5.01 for Windows (GraphPad Software, Arezzo, Italy) was used for statistical analysis. Statistical significance was tested with unpaired Students’ t test or one-way ANOVA. Statistically significant values of *p* are reported in the figure legends. Experiments were repeated three times in triplicates (if not differently stated) and data are presented as means ± S.D.

## 5. Conclusions

Our results demonstrate that the c10orf118 protein isolated from breast tumour cell lines has a full-size molecular weight of 130 kDa and is localized in the Golgi apparatus. It is highly synthesized by the low metastatic cells. Such a protein contributes to the crosstalk between cancer and stromal cells, as it induces the secretion of HA by the surrounding fibroblasts through the up regulation of the hyaluronan synthase 2 gene (HAS2). In MCF-7 cells, an autocrine effect of c10orf118 on HAS2, its antisense and CD44 was found. Moreover, expression analysis conducted on breast cancer patient specimens have confirmed the presence of c10orf118, whereas Kaplan-Meier analysis shows that it is positively associated with high overall survival. Further studies on the mechanism by which this Golgi-localized c10orf118 protein is secreted and induces HA-related enzymes are necessary in order to fully understand its autocrine and paracrine role in breast cancer microenvironment.

## Data Availability

The data presented in this study are available on request from the corresponding author.

## Supplementary Materials

The following are available online at https://www.mdpi.com/2072-6694/13/5/1105/s1, Figure S1: Peptides spectrum and predicted structure of c10orf118. (A) MALDI-TOF spectrum of peptides obtained from SDS-PAGE band excised and analysed as reported in Materials and Methods. (B) MASCOT Search results of the peptides matching the human uncharacterized protein c10orf118. (C) prediction of the protein 3D-structure by Phyre2 program, showing the presence of many alpha-helix as secondary structures. Figure S2: Full image of RT-PCR and western blot analysis. (A) RT-PCR analysis of the c10orf118 gene expression in 20 tissue specimens from breast cancer patients; (B) western blot analysis of the c10orf118 protein expression in 20 tissue specimens from breast cancer patients. Table S1: Information of different gene variants and protein isoforms of c10orf118, according to Ensembl bioinformatics program. Table S2: Predicted cellular localization of C10orf118.
