# Supplementary material for: The Secreted Protein C10orf118 Is a New Regulator of Hyaluronan Synthesis Involved in Tumour-Stroma Cross-Talk
Source: Cancers (Basel). 2021 Mar 5;13(5):1105. doi: 10.3390/cancers13051105 (PMC7961460; doi:10.3390/cancers13051105)
Supplement: Supplementary file 1 [file cancers-13-01105-s001.pdf]

cancers

Article

The Secreted Protein C10orf118 Is a New Regulator of Hyaluronan Synthesis Involved in Tumour-Stroma Cross-Talk

Ilaria Caon [1,†], Maria Luisa D'Angelo [1,†], Barbara Bartolini [1,†], Elena Caravà [1], Arianna Parnigoni [1], Flavia Contino [2], Patrizia Cancemi [2,3], Paola Moretto [1], Nikos K. Karamanos [4], Alberto Passi [1], Davide Vigetti [1], Evgenia Karousou [1,*] and Manuela Viola [1]

1 Department of Medicine and Surgery, University of Insubria, Via J.H. Dunant 5, 21100 Varese, Italy; i.caon@uninsubria.it (I.C.); marialuisadangelo@gmail.com (M.L.D.); bartolini.barbara@gmail.com (B.B.); e.carava@uninsubria.it (E.C.); a.parnigoni@uninsubria.it (A.P.); paola.moretto@uninsubria.it (P.M.); alberto.passi@uninsubria.it (A.P.); Davide.Vigetti@uninsubria.it (D.V.); manuela.viola@uninsubria.it (M.V.)
2 Department of Biological Chemical and Pharmaceutical Sciences and Technologies (STEBICEF), University of Palermo, 90128 Palermo, Italy; flavia.contino@unipa.it (F.C.); patrizia.cancemi@unipa.it (P.C.)
3 Centro di Oncobiologia Sperimentale (C.O.B.S.), Oncology Department, La Maddalena, 90146 Palermo, Italy
4 Biochemical Analysis and Matrix Pathobiology Research Group, Laboratory of Biochemistry, Department of Chemistry, University of Patras, 26110 Patras, Greece; n.k.karamanos@upatras.gr
* Correspondence: jenny.karousou@uninsubria.it
† These authors contributed equally to this work.

Citation: Caon, I.; D'Angelo, M.L.; Bartolini, B.; Carav, E.; Parnigoni, A.; Contino, F.; Cancemi, P.; Moretto, P.; Karamanos, N.K.; Passi, A.; et al. The Secreted Protein C10orf118 Is a New Regulator of Hyaluronan Synthesis Involved in Tumou-Stroma Cross-Talk. *Cancers* **2021**, *13*, 1105. https://doi.org/10.3390/cancers13051105

Academic Editor: Derek Radisky and Hamid Morjani

Received: 30 January 2021
Accepted: 26 February 2021
Published: 5 March 2021

Publisher's Note: MDPI stays neutral with regard to jurisdictional claims in published maps and institutional affiliations.

Copyright: © 2021 by the authors. Licensee MDPI, Basel, Switzerland. This article is an open access article distributed under the terms and conditions of the Creative Commons Attribution (CC BY) license (http://creativecommons.org/licenses/by/4.0/).

Supplementary

A

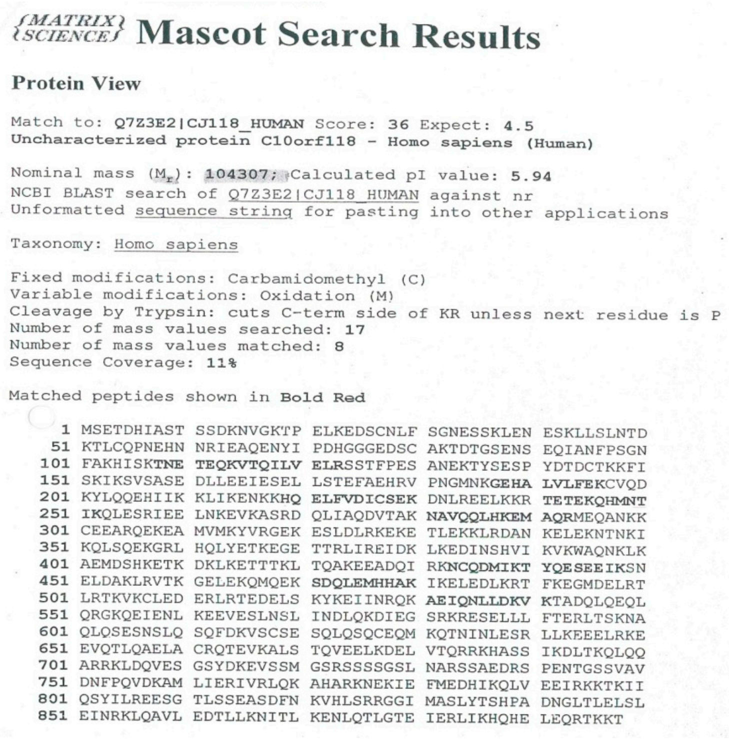

MATRIX SCIENCE **Mascot Search Results**

Protein View

Match to: **Q7Z3E2|CJ118_HUMAN** Score: **36** Expect: **4.5**
Uncharacterized protein C10orf118 - Homo sapiens (Human)

Nominal mass (M_r): **104307**; Calculated pI value: **5.94**
NCBI BLAST search of Q7Z3E2|CJ118_HUMAN against nr
Unformatted sequence string for pasting into other applications

Taxonomy: Homo sapiens

Fixed modifications: Carbamidomethyl (C)
Variable modifications: Oxidation (M)
Cleavage by Trypsin: cuts C-term side of KR unless next residue is P
Number of mass values searched: **17**
Number of mass values matched: **8**
Sequence Coverage: **11%**

Matched peptides shown in **Bold Red**

1 MSETDHIAST SSDKNVGKTP ELKEDSCNLF SGNESSKLEN ESKLLSLNTD
51 KTLCQPNEHN NRIEAQENYI PDHGGGEDSC AKTDTGSENS EQIANFPSGN
101 FAKHISK**TNE** **TEQKVTQILV** **ELR**SSTFPES ANEKTYSESP YDTDCTKKFI
151 SKIKSVSASE DLLEEIESEL LSTEFAEHRV PNGMNK**GEHA** **LVLFEK**CVQD
201 KYLQQEHIIK KLIKENKK**HQ** **ELFVDICSEK** DNLREELKKR **TETEKQHMNT**
251 **IK**QLESRIEE LNKEVKASRD QLIAQDVTAK **NAVQQLHKEM** **AQR**MEQANKK
301 CEEARQEKEA MVMKYVRGEK ESLDLRKEKE TLEKKLRDAN KELEKNTNKI
351 KQLSQEKGRL HQLYETKEGE TTRLIREIDK LKEDINSHVI KVKWAQNKLK
401 AEMDSHKETK DKLKETTTKL TQAKEEADQI RK**NCQDMIKT** **YQESEEIK**SN
451 ELDAKLRVTK GELEKQMQEK **SDQLEMHHAK** IKELEDLKRT FKEGMDELRT
501 LRTKVKCLED ERLRTEDELS KYKEIINRQK **AEIQNLLDKV** KTADQLQEQL
551 QRGKQEIENL KEEVESLNSL INDLQKDIEG SRKRESELLL FTERLTSKNA
601 QLQSESNSLQ SQFDKVSCSE SQLQSQCEQM KQTNINLESR LLKEEELRKE
651 EVQTLQAELA CRQTEVKALS TQVEELKDEL VTQRRKHASS IKDLTKQLQQ
701 ARRKLDQVES GSYDKEVSSM GSRSSSSGSL NARSSAEDRS PENTGSSVAV
751 DNFPQVDKAM LIERIVRLQK AHARKNEKIE FMEDHIKQLV EEIRKKTKII
801 QSYILREESG TLSSEASDFN KVHLSRRGGI MASLYTSHPA DNGLTLELSL
851 EINRKLQAVL EDTLLKNITL KENLQTLGTE IERLIKHQHE LEQRTKKT

B

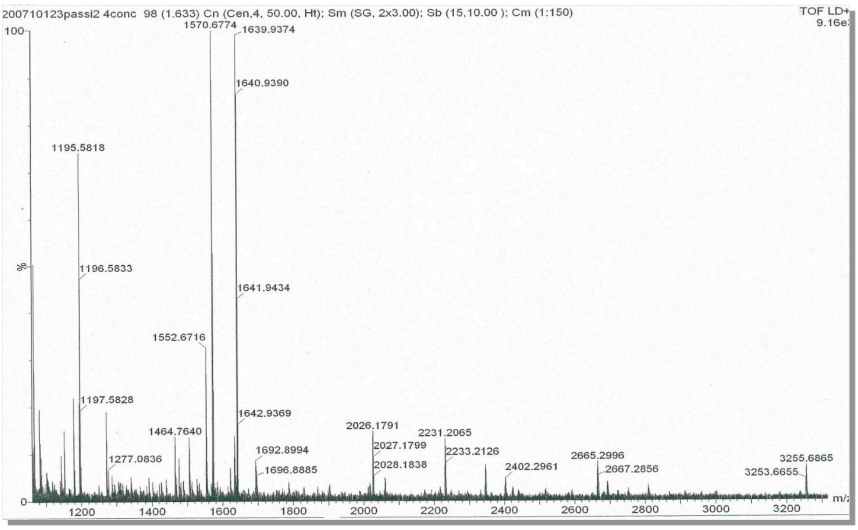

C

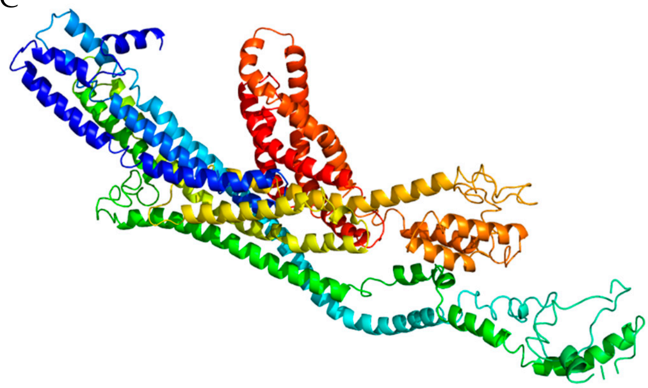

Figure S1. (**A**) MALDI-TOF spectrum of peptides obtained from SDS-PAGE band excised and analysed as reported in Materials and Methods. (**B**) MASCOT Search results of the peptides matching the human uncharacterized protein c10orf118. (**C**) Prediction of the protein 3D-structure by Phyre2 program, showing the presence of many alpha-helix as secondary structures.

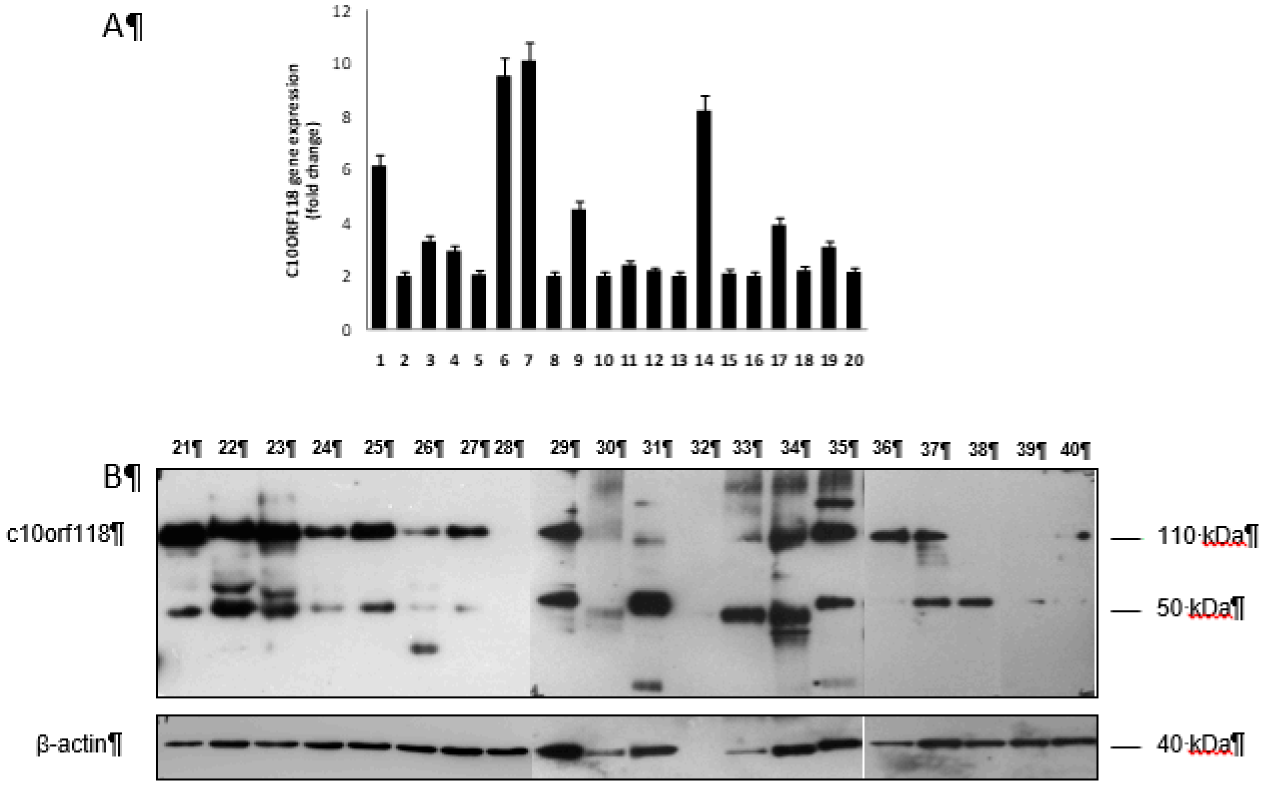

Figure S2. A. RT-PCR analysis of the c10orf118 gene expression in 20 tissue specimens from breast cancer patients; B. western blot analysis of the c10orf118 protein expression in 20 tissue specimens from breast cancer patients.

Table S1. Information of different gene variants and protein isoforms of c10orf118, according to Ensembl bioinformatics program.

Name	Transcript ID	bp	Protein	Translation ID	Biotype
CCDC186-006	ENST00000369287	7245	898aa	ENSP00000358293	Protein coding
CCDC186-003	ENST00000369285	2035	211aa	ENSP00000358291	Protein coding
CCDC186-002	ENST00000369286	1928	211aa	ENSP00000358292	Protein coding
CCDC186-001	ENST00000428953	1576	436aa	ENSP00000415344	Protein coding
CCDC186-004	ENST00000497592	661	No protein	-	Processed transcript
CCDC186-005	ENST00000490661	477	No protein	-	Retained intron

Table S2. Cellular localization of c10orf118 predicted by ComPPI computational program (http://comppi.linkgroup.hu/).

Major Localization	Minor Localization	Localization Score	Experiment Type	Source Database
Cytosol	Cytoplasm (GO:0005737)	0.91	Predicted: pTarget method	LOCATE
Cytosol	Cytoplasm (GO:0005737)	0.91	Predicted: PAML algorithm	PA-GOSUB
Nucleus	Nucleus (GO:0005634)	0.7	Predicted: SVM decision tree	eSLDB
Secretory-pathway	Golgi apparatus (GO:0005794)	0.94	Predicted: PAML algorithm	PA-GOSUB
**Secretory-pathway**	**Golgi apparatus (GO:0005794)**	**0.94**	**Experimental: annotated protein expression (APE)**	**The Human Protein Atlas**
